# Dapagliflozin promotes angiogenesis in hindlimb ischemia mice by inducing M2 macrophage polarization

**DOI:** 10.3389/fphar.2023.1255904

**Published:** 2023-09-21

**Authors:** Heng Yang, Wanqi Lan, Wu Liu, Tingtao Chen, Yanhua Tang

**Affiliations:** ^1^ Department of Cardiovascular Surgery, The Second Affiliated Hospital of Nanchang University, Nanchang, China; ^2^ The Second Clinical Medical College of Nanchang University, Nanchang, China; ^3^ The Institute of Translational Medicine, Nanchang University, Nanchang, China

**Keywords:** critical limb ischemia, dapagliflozin, angiogenesis, macrophage polarization, HUVECs

## Abstract

Critical limb ischemia (CLI) is associated with a higher risk of limb amputation and cardiovascular death. Dapagliflozin has shown great potential in the treatment of cardiovascular disease. However, the effects of dapagliflozin on CLI and the underlying mechanisms have not been fully elucidated. We evaluated the effect of dapagliflozin on recovery from limb ischemia using a mouse model of hindlimb ischemia. The flow of perfusion was evaluated using a laser Doppler system. Tissue response was assessed by analyzing capillary density, arterial density, and the degree of fibrosis in the gastrocnemius muscle. Immunofluorescence and Western blot were used to detect the expression of macrophage polarization markers and inflammatory factors. Our findings demonstrate the significant impact of dapagliflozin on the acceleration of blood flow recovery in a hindlimb ischemia mouse model, concomitant with a notable reduction in limb necrosis. Histological analysis revealed that dapagliflozin administration augmented the expression of key angiogenic markers, specifically CD31 and α-SMA, while concurrently mitigating muscle fibrosis. Furthermore, our investigation unveiled dapagliflozin’s ability to induce a phenotypic shift of macrophages from M1 to M2, thereby diminishing the expression of inflammatory factors, including IL-1β, IL-6, and TNF-α. These effects were partially mediated through modulation of the NF-κB signaling pathway. Lastly, we observed that endothelial cell proliferation, migration, and tube-forming function are enhanced *in vitro* by utilizing a macrophage-conditioned medium derived from dapagliflozin treatment. Taken together, our study provides evidence that dapagliflozin holds potential as an efficacious therapeutic intervention in managing CLI by stimulating angiogenesis, thereby offering a novel option for clinical CLI treatment.

## 1 Introduction

Critical limb ischemia (CLI) is the most severe manifestation of peripheral arterial disease (PAD), primarily caused by atherosclerosis, affecting millions of individuals worldwide (Farber and Eberhardt, 2016; Conte and Vale, 2018). Patients with CLI typically experience rest pain, necrosis, and gangrene, with advancing disease stages culminating in myocardial infarction, stroke, amputation, and death, which brings a significant burden to patients and society (Shishehbor et al., 2016). Despite the availability of several therapeutic interventions for CLI, their effectiveness remains unsatisfactory (Hassanshahi et al., 2019). Therefore, it is critical to identify a viable treatment strategy or drug capable of retarding disease progression and enhancing patient outcomes.

Conventional treatment strategies for CLI typically consist of surgical and pharmacological interventions (Teraa et al., 2016). Surgical interventions, such as bypass surgery and intravascular treatment, aim to restore blood perfusion and prevent further injury to ischemic tissue. However, up to half of patients may not be suitable for surgical interventions due to various factors, such as comorbidity, adverse anatomy, and a wide range of diseased vessels (Shammas et al., 2012; Lichtenberg et al., 2018). Pharmacological treatment primarily involves managing and preventing risk factors associated with CLI, such as hypertension, hyperlipidemia, and thrombosis, but is limited in its ability to restore tissue perfusion and may result in hepatic and renal side effects as well as high bleeding risk (Xing et al., 2021). Therapeutic angiogenesis, which aims to promote new vessel formation from pre-existing blood vessels to increase blood perfusion in ischemic limbs, is a promising strategy for overcoming the limitations of conventional CLI treatment (Zampetaki et al., 2008). Gao et al. demonstrated that mesenchymal stem cells could promote angiogenesis in the ischemic limbs of CLI mice by affecting macrophage polarization and inflammation (Song et al., 2020). Additionally, Slobodkina et al. have constructed a plasmid expressing VEGF165 and HGF, which has been shown to promote tube formation in HUVECs and improve ischemic tissue perfusion in CLI mice (Slobodkina et al., 2020). Stem cell therapy, growth factor therapy, and gene therapy are among the current therapeutic angiogenesis strategies (Veith et al., 2019). However, the effectiveness of these treatment strategies in clinical trials remains to be improved (Iyer and Annex, 2017).

The development of new drugs is a challenging process that is time-consuming, expensive, complex, and frequently fails. Drug repurposing, a promising strategy that involves finding new uses or indications for existing drugs, has gained traction among researchers and pharmaceutical companies due to its low cost and short development time (Nosengo, 2016). One notable example is aspirin, which was initially used to treat fever and body pain but is now widely utilized to prevent cardiovascular diseases due to its antithrombotic properties (Montinari et al., 2019). Dapagliflozin, a novel antidiabetic drug, selectively inhibits the sodium-glucose co-transport protein 2 (SGLT2i) in the proximal renal tubule, thereby increasing urinary glucose excretion and preventing renal glucose reabsorption (Thomas and Cherney, 2018). A large multicenter randomized controlled trial (DAPA-HF), including 4,744 patients with heart failure, recently discovered that dapagliflozin treatment reduced the risk of worsening heart failure (9.7% vs. 13.4%) and the risk of death from cardiovascular causes (9.6% vs. 11.5%) in both diabetic and non-diabetic patients (McMurray et al., 2019). A study has shown that dapagliflozin effectively promotes angiogenesis and restores blood perfusion to ischaemic limbs in diabetic HLI mice (Nugrahaningrum et al., 2020). However, Uthman et al. (Uthman et al., 2019) investigated the effects of dapagliflozin on human coronary artery endothelial cells (HCAECs) and human umbilical vein endothelial cells (HUVECs) that were stimulated with tumor necrosis factor-alpha (TNF-α). The study found that dapagliflozin inhibited the production of reactive oxygen species (ROS) in endothelial cells and restored nitric oxide (NO) bioavailability. Similarly, Gaspari et al. (Gaspari et al., 2018) discovered that dapagliflozin reduced inflammatory cytokines and improved endothelial diastolic function. These results suggest that dapagliflozin may be vital in enhancing cardiovascular function and promoting angiogenesis through mechanisms independent of its hypoglycemic effects. However, whether dapagliflozin promotes post-ischemic angiogenesis in non-diabetic states and the underlying mechanisms have not been elucidated.

In this study, we administered dapagliflozin to non-diabetic mice and established a hindlimb ischemia (HLI) model to determine the role of dapagliflozin in hind limb recovery after CLI. Our results revealed that treatment with dapagliflozin improved blood flow recovery in mice with hindlimb ischemia, which may indirectly promote angiogenesis by affecting the polarization of macrophages.

## 2 Materials and methods

### 2.1 Animals

Eight-week-old male C57BL/6 mice weighing 20–26 g were procured from Jie Si Jie Laboratory Animal Corp (Shanghai, China). The mice were kept in an SPF environment, following a standard light/dark cycle of 12 h, with *ad libitum* access to food and water. The study was conducted at Nanchang University, China, following institutional guidelines and authorized by the Animal Ethics Committee, with an ethical institution number of SYXK 2021-0001, and an animal experimentation ethics number of NCULAE-20230610002. The welfare of the mice was ensured following the updated 2011 eighth edition of the Guide for the Care and Use of Laboratory Animals from the National Institutes of Health.

### 2.2 Mouse hind limb ischemia model

Mice were subjected to oral administration of dapagliflozin (1 mg/kg/day, Sigma-Aldrich Co. St. Louis, MO) or saline for 2 weeks. Subsequently, ten-week-old mice were anesthetized by inhalation of isoflurane. The surgical site was prepared by shaving and cleaning the fur with ethanol, and the femoral artery was isolated from the saphenous nerve and vein. Ligation of the femoral artery was conducted at the proximal profunda femoris and epigastric arteries and distal popliteal fossa, while the right limb served as a control. The surgical site was then sutured.

### 2.3 Laser Doppler perfusion imaging

After ischemic surgery, laser Doppler perfusion imaging (PeriScan PIM 3, Perimed) was performed on days 0, 3, 7, 14, and 21 to evaluate blood flow in the hindlimbs. The mice were anesthetized with isoflurane (2%–4%) and maintained on a heating pad at 37°C for 3 minutes. The blood flow was displayed using different colored pixels corresponding to changes in laser frequency. The perfusion ratio between the ischemic and non-ischemic hindlimbs was calculated by determining the mean pixel value.

### 2.4 Necrosis score and limb motor function score

Tissue damage and hindlimb functionality were evaluated in mice subjected to the hindlimb ischemia (HLI) model on the 14th day postoperatively. The severity of necrosis was assessed using a scoring system, wherein a score of 0 denoted the absence of necrosis in the ischemic limb, a score of 1 indicated necrosis limited to the toe, a score of 2 represented necrosis extending to the dorsum of the foot, a score of 3 corresponded to necrosis extending to the crus, and a score of 4 indicated necrosis extending to the mid-tibia or complete limb necrosis. To evaluate limb motor function, a scoring system was employed. Mice exhibiting no usage of the limb were assigned a score of 1. Mice demonstrating limb usage but no utilization of the foot were assigned a score of 2. Restricted foot usage led to a score of 3, while mice displaying foot usage but no toe movement received a score of 4. Mice with unrestricted limb usage were assigned the highest score of 5 (Ma et al., 2021).

### 2.5 Immunohistochemistry assay

The gastrocnemius muscles were collected on day 21 post-surgery and sliced into 8-mm-thick paraffin-embedded sections. They were dewaxed and rehydrated before undergoing an antigen retrieval process. Subsequently, the sections were incubated with antibodies against a-SMA (1:100 dilution, ab5694, Abcam, Cambridge, United Kingdom) and CD31 (1:1000 dilution, ab182981, Abcam, Cambridge, United Kingdom) overnight at 4°C. After several washes with TBST, the sections were treated with secondary HRP-labeled antibodies (1:200 dilution) for 2 h at room temperature. Finally, the CD31 and a-SMA staining was semiquantitatively evaluated using ImageJ software. Two or four fields of view were examined in each section and the mean capillary density or arterioles density values for each field of view were recorded.

### 2.6 Masson’s trichrome staining

To quantify perivascular fibrosis in the gastrocnemius muscles, a fixation step using 4% paraformaldehyde was followed by paraffin embedding. Masson trichrome staining was conducted to visualize the fibrosis, and a digital imaging system was utilized to measure the fibrotic area. This was then represented as a percentage of the total tissue area.

### 2.7 Immunofluorescence

The paraffin-embedded sections were incubated with primary antibodies for CD68 (1:50 dilution, ab283654, Abcam, Cambridge, United Kingdom), iNOS (1:200 dilution, ab178945, Abcam, Cambridge, United Kingdom), and CD206 (1:1000 dilution, ab64693, Abcam, Cambridge, United Kingdom) overnight at 4°C, followed by treatment with appropriate secondary antibodies (1:400 dilution) and counterstaining with DAPI. The labeled cells were examined under fluorescence microscopy.

### 2.8 Cell culture and treatment

Bone-marrow-derived macrophages (BMDMs, 1 × 10^6^ cells/each well in 6-well plate) were collected from 6 to 8-week-old mice via flushing of the femur and tibia, and were cultured in DMEM (Gibco, New York, United States) medium supplemented with 100 ng/mL M-CSF (Sigma-Aldrich Co. St. Louis, MO) for 5 days. After 36 h of treatment with dapagliflozin, macrophages were subjected to an exposure of 100 ng/mL LPS (Sigma-Aldrich Co. St. Louis, MO) for 12 h, following which they were deemed the conditioned medium (CM) group. Macrophages that were exposed to 100 ng/mL LPS for 12 h without any prior treatment were categorized as Con group macrophages. The resulting CM from these macrophage cells was used to replace the medium for the subsequent incubation of HUVECs (Cyagen Biosciences).

### 2.9 Cell proliferation

To assess cell proliferation, HUVECs were exposed to a CM produced by macrophage cells for 36 h, with medium changes every 12 h. After pretreatment, the HUVECs were subjected to hypoxia for 36 h, and a 2-h treatment with 20 μM 5-ethynyl-2′-deoxyuridine (EdU, Beyotime, Shanghai, China) was performed at 37°C to measure cell proliferation. The nuclei of the proliferated cells were stained green, and the nuclei of all cells were counterstained with DAPI. The quantification of cell proliferation was determined by calculating the ratio of EdU + cells to the total number of cells using ImageJ.

### 2.10 Cell migration assay

Prior to performing the scratch assay and Transwell Migration Assay, HUVECs were treated with a CM for 36 h. Cells were seeded into 6-well plates and grown to 95% confluence, and then scratch wound was induced in a monolayer of cells using a p10 pipette tip, followed by cultivation in a sterilized hypoxic incubator for 48 h using a serum-free medium. The healing process of the wound was assessed by calculating the reduction in the wound gap utilizing the ImageJ software.

After the treatment, the HUVECs (1×10^5^/each well) were suspended in serum-free DMEM and were seeded into Transwell inserts (Corning, NJ), followed by exposure to a hypoxic environment for 8 h. A solution consisting of 5% serum in DMEM was utilized as the attractant, which was placed in the lower chamber of the Transwell insert. A 4% formaldehyde fixative and crystal violet staining were applied to the cells that migrated to the bottom surface of the membrane, whereas the cells remaining in the upper compartment were removed using cotton swabs.

### 2.11 Matrigel-based tube formation assay

For the tube formation assay, HUVECs were subjected to the same treatment conditions used for the proliferation assay for 36 h. The 24-well plates and tips were precooled at −20°C before the start of the experiments, and the plates were coated with Matrigel (BD Biosciences, New Jersey, United States) and incubated at 37°C for 30 min until the Matrigel solidified. The cells (1×10^5^/each well) were then added to the plate and incubated in a sterilized hypoxic incubator for 6 h. The resulting capillary-like structures were visualized using a microscope and quantified using ImageJ software.

### 2.12 Western blotting

Protein from the cells or tissue was extracted using an ice-cold RIPA buffer, and the concentration of proteins was quantified by a BCA kit (Invitrogen, California, United States). Afterward, around 20 μg of protein was boiled for 5 min at 98°C and subjected to SDS-polyacrylamide gel electrophoresis using either a 12% or 15% gel. The separated proteins were transferred onto PVDF (Millipore, Massachusetts, United States) membranes, followed by a blocking step using a 5% BSA (Solarbio Science & Technology, Beijing, China) solution for 120 min at room temperature. Primary antibodies were incubated with the PVDF membranes overnight at 4°C, followed by washing with TBST and incubation with secondary antibodies. Finally, the protein on the PVDF membranes was visualized using enhanced chemiluminescence (ECL Invitrogen, California, United States) solution. Anti-IL-1β (1:1000 dilution, 29530-1-AP, Proteintech, Wuhan, China), anti-IL-6 (1:1000 dilution, 12912, CST Danvers, MA, United States), anti-TNF-α (1:1000 dilution, 17590-1-AP, Proteintech, Wuhan, China), anti-iNOS (1:1000 dilution, ab178945, Abcam, Cambridge, United Kingdom), anti-Arg-1 (1:1000 dilution, 93668, CST Danvers, MA, United States), anti-phosphorylated-NF-κB (1:1000 dilution, 3034, CST Danvers, MA, United States), anti-NF-κB (1:1000 dilution, 3033, CST Danvers, MA, United States), anti-phosphorylated-IκBα (1:1000 dilution, 2895, CST Danvers, MA, United States), anti-IκBα (1:1000 dilution, 4,814, CST Danvers, MA, United States), and anti-β-actin (1:1000 dilution, 4,970, CST Danvers, MA, United States) antibodies were utilized in the experiment.

### 2.13 Statistical analysis

The present investigation employed GraphPad Prism 8 software to perform statistical analysis, with data presented as mean ± SD. The t-tests or non-parametric tests were conducted to compare the means of the two groups. Moreover, one-way ANOVA was utilized to compare the means among multiple groups, and the Bonferroni *post hoc* test was performed to evaluate the significance between the groups. The significance was determined at a threshold of *p* < 0.05.

## 3 Results

### 3.1 Dapagliflozin improves blood flow recovery and reduces necrosis in ischemic limbs

**FIGURE 1:**
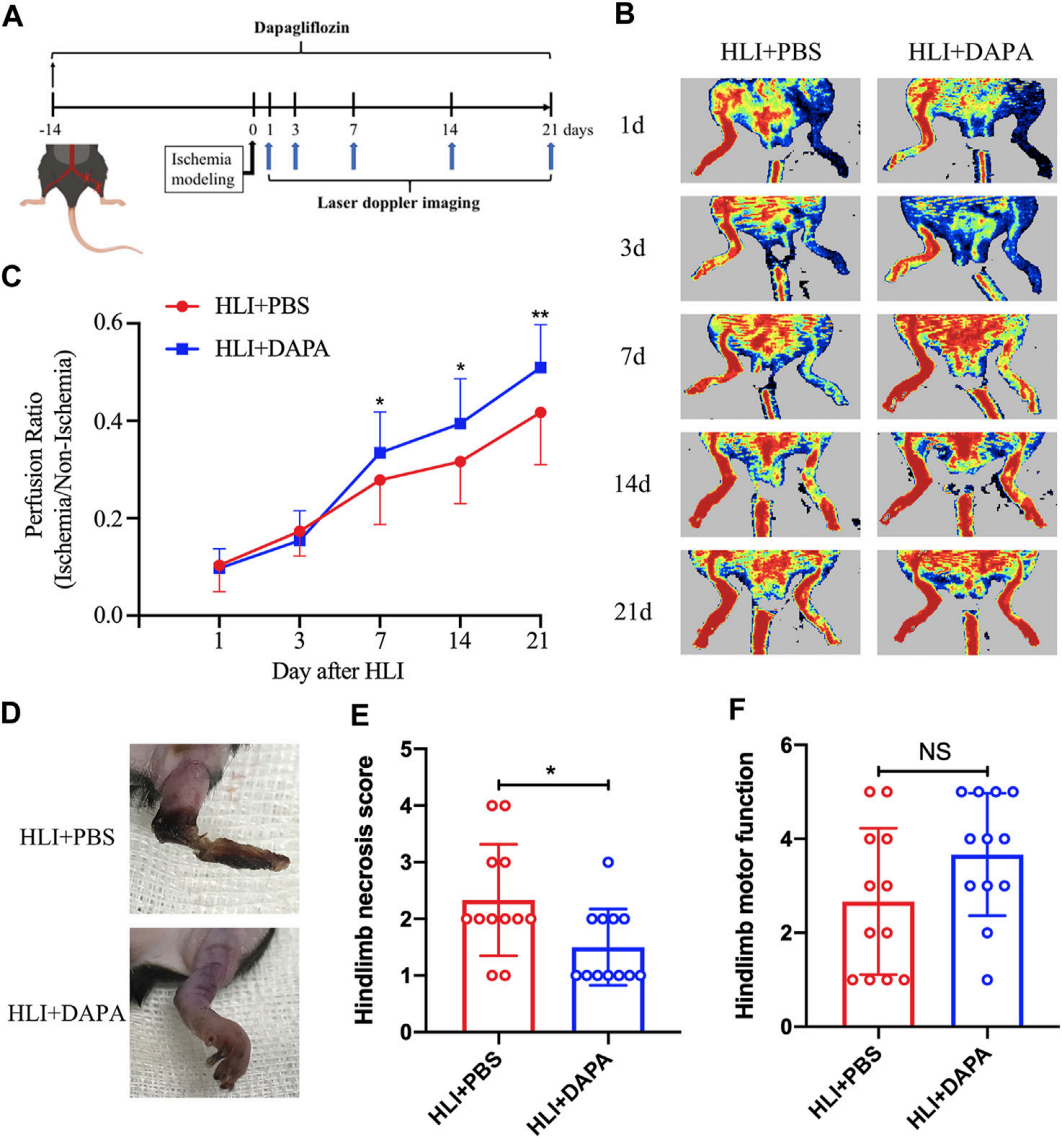
Dapagliflflozin improves blood flow recovery and reduces necrosis in ischemic limbs following HLI. **(A)** Schematic of schedule of the whole experiment. **(B)** Representative Laser Doppler perfusion imaging showed dynamic changes in blood perfusion in limb ischemia at days 0, 3, 7, 14, and 21 after HLI. **(C)** Quantitative analysis of blood flow is expressed as a ratio of left to right limb perfusion. **(D)** Representative images of ischemic limbs in mice at 5 days postoperatively. **(E)** A semi-quantitative analysis of the ischemic limb necrosis score. **(F)** A semi-quantitative analysis of the limb motor function score in mice. NS indicates no significance, *n* = 12. Statistical comparisons were made using a two-way ANOVA, followed by Sidak’s multiple comparisons post-tests **(C)** or an unpaired t-test **(E, F)**, *p < 0.05, **p < 0.01.

To assess the effect of dapagliflozin on hindlimb ischemic disease, C57BL/6 mice were pretreated with dapagliflozin for 2 weeks, and then the HLI model was induced by ligating the unilateral femoral artery of mice. Laser Doppler imaging assessed perfusion recovery on days 1, 3, 7, 14, and 21 after the operation (Figure 1A). The results showed a significant improvement in blood flow recovery in the dapagliflozin-treated group after the surgical procedure compared to the PBS group (Figure 1B; C, Two-way ANOVA, *p* < 0.05). In addition, Tissue damage and limb motor function were scored and statistically analysed by unpaired *t*-test in both groups, which revealed that the gangrene and limb necrosis scores were strikingly higher in the PBS-treated groups than in the DAPA-treated group (HLI + PBS vs. HLI + DAPA = 2.33 vs. 1.54, unpaired *t*-test, *p* < 0.05) (Figure 1D; E), but there was no remarkable difference in limb motor function scores (Figure 1F).

### 3.2 Effects of dapagliflozin on angiogenesis and anti-fibrosis in ischemic muscle

**FIGURE 2:**
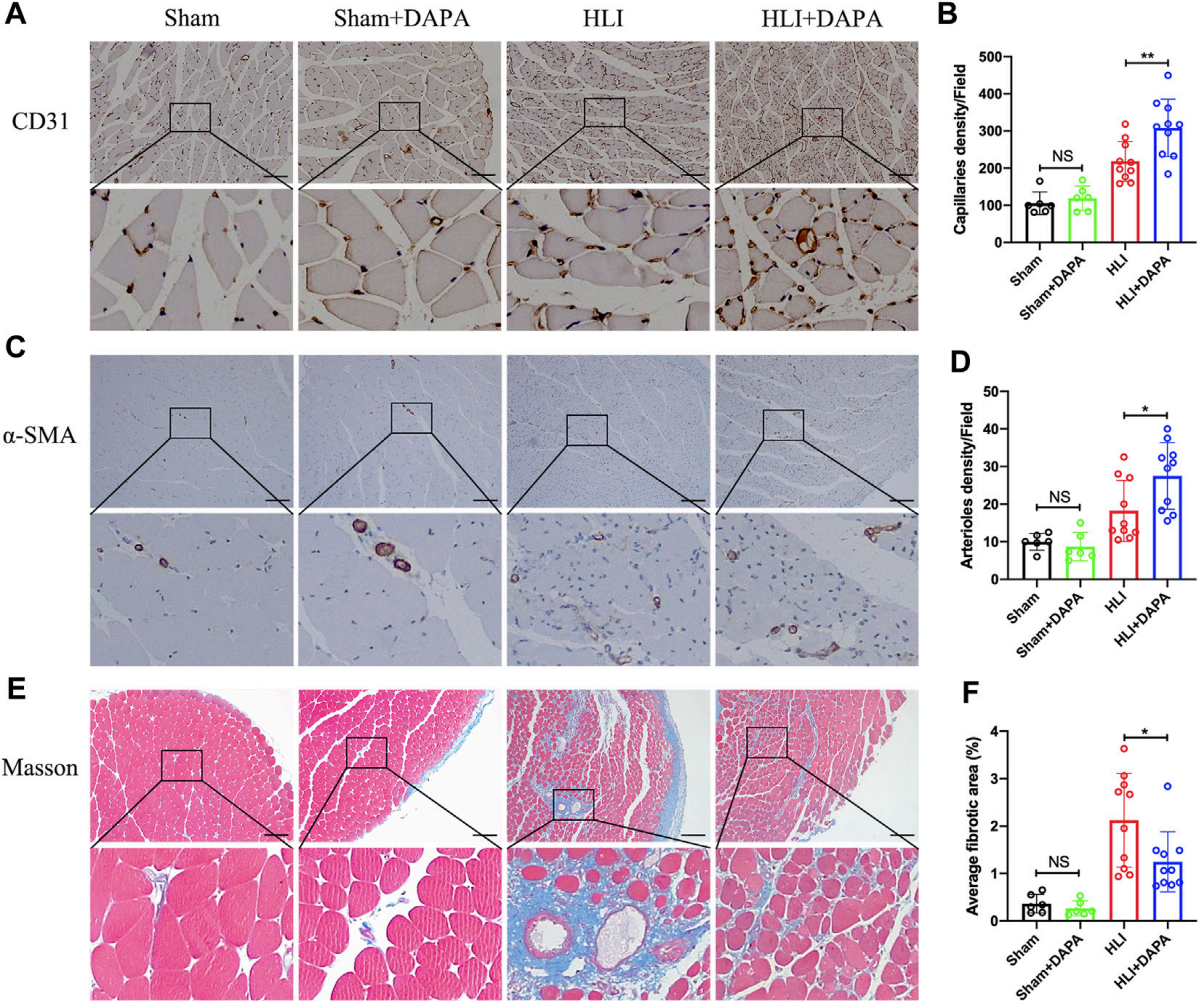
Histological evaluation of angiogenesis and fibrosis in mouse gastrocnemius muscle. **(A, B)** Representative images **(A)** and quantification **(B)** of anti-CD31 immunohistochemical staining of mice gastrocnemius muscle 21 days after HLI. Scale bar = 100 μm. **(C, D)** Representative anti-α smooth muscle actin (α-SMA) immunohistochemical staining **(C)** of gastrocnemius muscle sections and quantitative analysis **(D)** of the α-SMA + arteriole density. Scale bar = 250 μm. **(E, F)** Masson-trichrome (Masson) staining **(E)** and quantitative analysis **(F)** for identification of fibrotic area (blue) in gastrocnemius muscle tissue. Scale bar = 250 μm. NS indicates no significance, n = 6–10, Multiple group comparisons were made using one-way ANOVA, followed by Bonferroni *post hoc* test, *p < 0.05, **p < 0.01.

The gastrocnemius muscle tissue of mice was then collected on the 21st day after the operation to evaluate the effect of dapagliflozin on angiogenesis. Immunohistochemical analysis was used to evaluate the expression of CD31 and α-SMA in muscle tissue as characteristic markers for capillary and arteriole. The results showed that the capillary (HLI + PBS vs. HLI + DAPA = 217.97 vs. 308.03, one-way ANOVA, *p* < 0.01) (Figure 2A; B) and arteriole (HLI + PBS vs. HLI + DAPA = 18.23 vs. 27.48, one-way ANOVA, *p* < 0.05) (Figure 2C; D) densities in ischemic muscles were significantly increased in the HLI + DAPA group mice than in those administered the PBS control. Moreover, a Masson’s trichrome staining of gastrocnemius muscles was carried out to assess the degree of fibrosis (Figure 2E), and the results revealed that the fibrotic area was remarkably smaller in the HLI + DAPA group on day 21 after the operation (HLI + PBS vs. HLI + DAPA = 2.12% vs. 1.25%, one-way ANOVA, *p* < 0.05) (Figure 2F).

### 3.3 Dapagliflozin promoted M2 macrophage polarization

**FIGURE 3:**
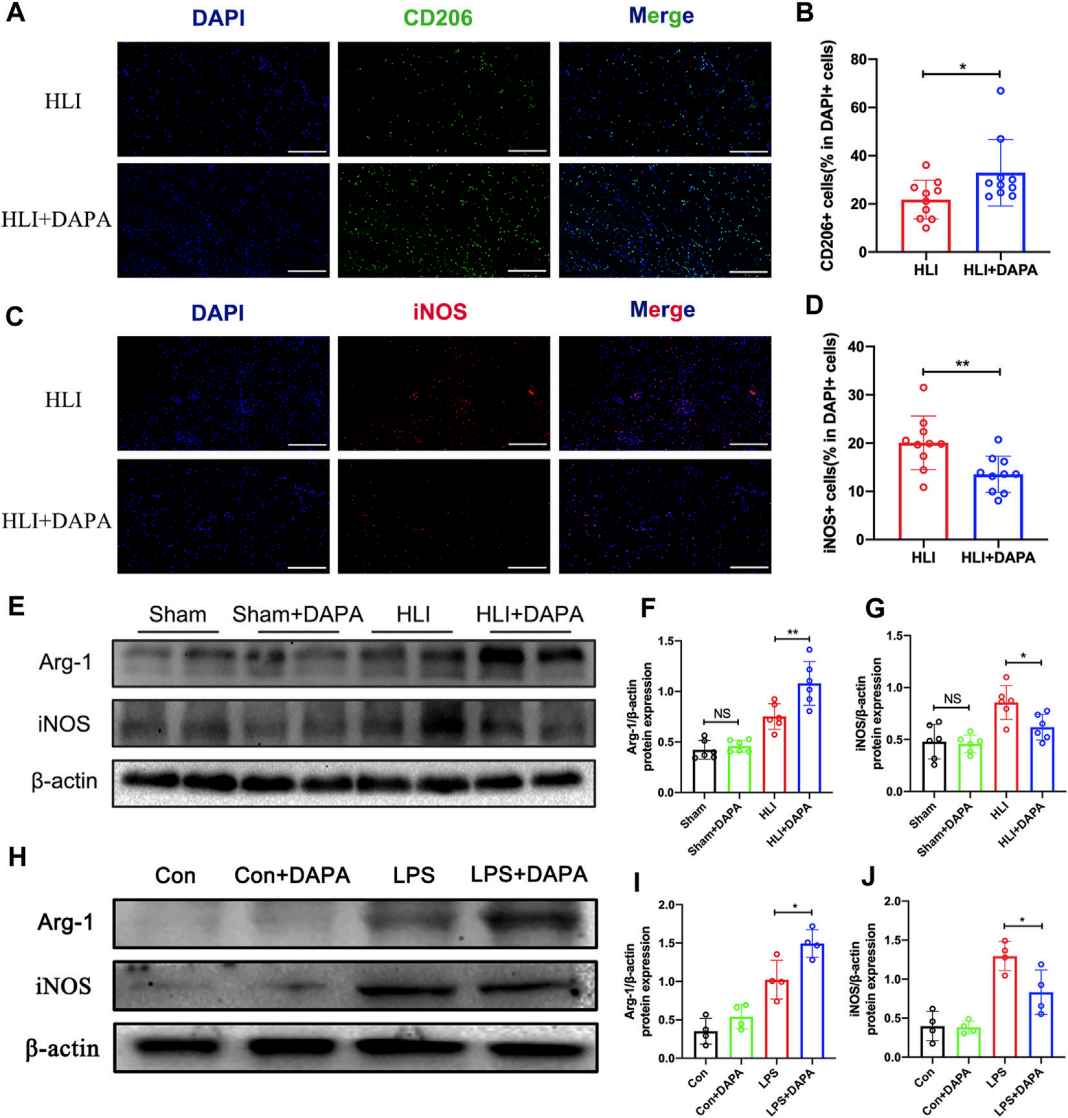
The effect of dapagliflozin on the polarization state of macrophages. **(A)** Representative images of CD206 and DAPI staining in the ischemic gastrocnemius muscle. **(B)** Quantitative analysis of the M2 macrophages was calculated as the ratio of CD206+ cells and DAPI + cells. Scale bar = 200 μm. **(C)** Representative images of iNOS and DAPI staining in the ischemic gastrocnemius muscle. **(D)** Quantitative analysis of the M1 macrophages was calculated as the ratio of iNOS + cells and DAPI + cells. Scale bar = 200 μm. (E, F, and **(G)** Representative Western blotting images **(E)** and summary densitometry graph illustrating expression of Arg-1 **(F)** and iNOS **(G)** in the ischemic gastrocnemius muscle at 21 days postoperatively. **(H–J)** Representative Western blotting images **(H)** and summary densitometry graph illustrating expression of Arg-1 **(I)** and iNOS **(J)** in mouse bone marrow-derived macrophages (BMDM) stimulated with or without LPS *in vitro*. n = 6–10 **(A–D)**; n = 6–10 **(E–J)**, Statistical comparisons were made using Mann–Whitney test **(C)** or an unpaired *t*-test **(D,F,G,I and J)**, *p < 0.05, **p < 0.01.

Macrophages play a critical role in angiogenesis after an ischemic attack (Fu et al., 2023). We evaluated macrophage infiltration in mouse gastrocnemius muscle tissue collected on day five after HLI. Analysis of immunofluorescence staining showed no significant differences in total macrophage numbers defined by CD68-positive between the PBS and dapagliflozin-treated groups (Supplementary Figures S1A, B). Interestingly, the expression levels of CD206 (the marker of M2 macrophages) were significantly higher in ischemic muscle from DAPA-treated mice than in PBS-treated mice (HLI vs. HLI + DAPA = 21.78% vs. 32.92%, unpaired *t*-test, *p* < 0.05) (Figure 3A; B). Correspondingly, iNOS (the marker of M1 macrophages) immunofluorescent staining showed significantly lower M1 macrophage density in the HLI + DAPA group (HLI vs. HLI + DAPA = 20.06% vs. 13.54%, Mann–Whitney test, *p* < 0.01) (Figure 3C; D). These results were further confirmed by Western blot analysis (Figure 3E), which showed that dapagliflozin treatment significantly decreased iNOS expression (HLI vs. HLI + DAPA = 0.86 vs. 0.62, unpaired *t*-test, *p* < 0.05) and increased the protein expression level of Arg-1 (HLI vs. HLI + DAPA = 0.75 vs. 1.08, unpaired *t*-test, *p* < 0.01), a marker highly expressed in M2 macrophages (Figure 3F; G).

Next, mouse bone marrow-derived macrophages (BMDMs) were utilized *in vitro* experiments. The protein expression levels of iNOS and Arg-1 in BMDMs were evaluated following treatment with dapagliflozin at varying concentrations and treatment times. Western blot analysis demonstrated that the protein expression of Arg-1 was upregulated as dapagliflozin treatment time (Supplementary Figures S2A, B) and concentration (Supplementary Figures S2C, D) increased, while the expression of iNOS was downregulated. Based on these results, an effective concentration of 10 μM and a treatment time of 36 h was used in subsequent experiments (Figure 3H). The results showed that dapagliflozin significantly decreased the protein expression of iNOS (LPS vs. LPS + DAPA = 1.29 vs. 0.83, unpaired *t*-test, *p* < 0.05) and increased the expression of Arg-1 (LPS vs. LPS + DAPA = 1.02 vs. 1.49, unpaired *t*-test, *p* < 0.05) in LPS-stimulated BMDMs (Figure 3I; J).

### 3.4 The effect of dapagliflozin on macrophage M2 polarization may be achieved by inhibiting the NF-κB pathway

**FIGURE 4:**
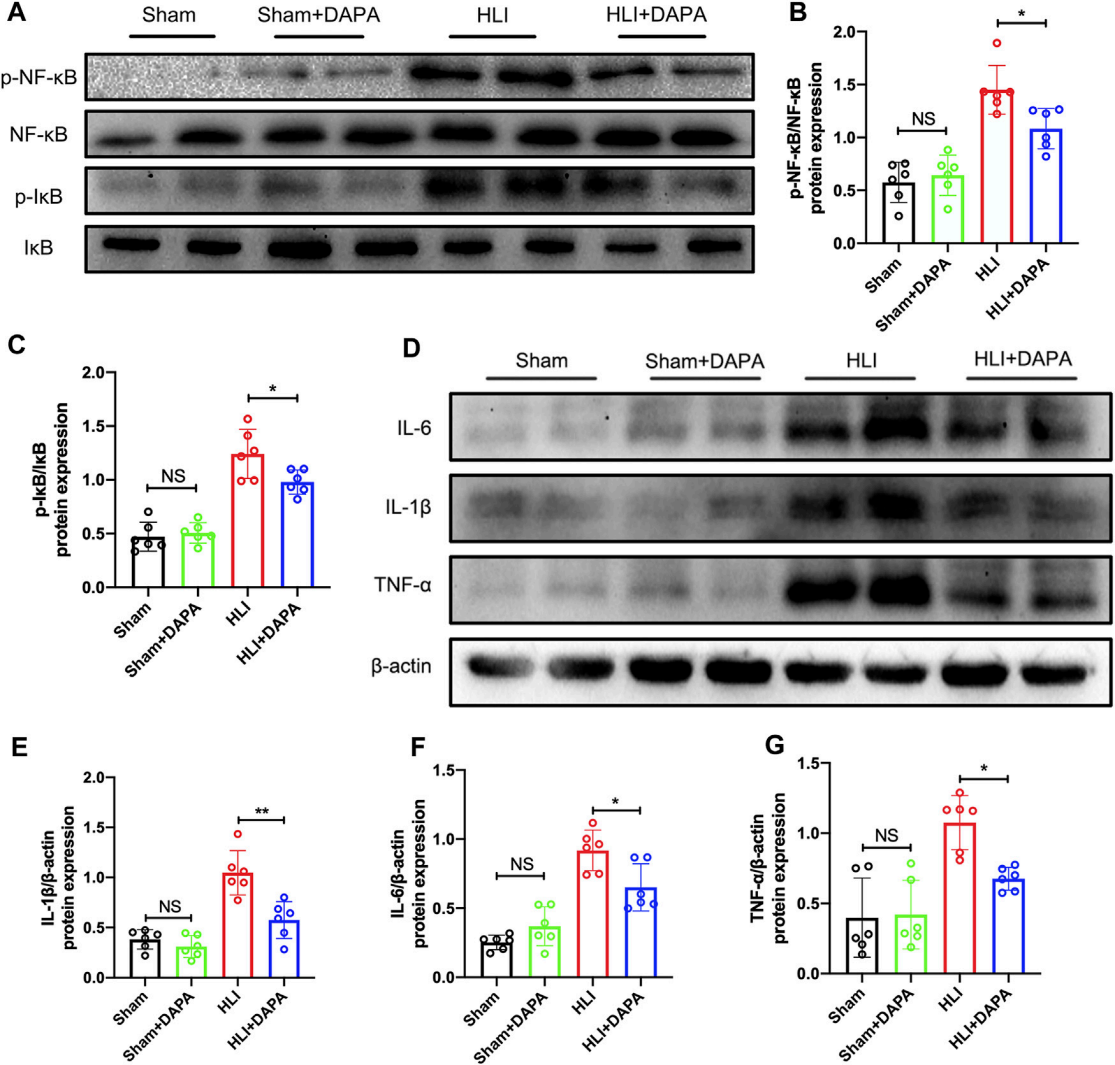
The effect of dapagliflozin on macrophage M2 polarization may be achieved through the NF-κB pathway. **(A–C)** The protein levels of IκB, phosphorylated- IκB, NF-κB, and phosphorylated-NF-κB in the ischemic gastrocnemius muscle were examined using Western blotting. **(A)** Representative Western blotting images and **(B, C)** summary densitometry graphs were shown. **(D)** Representative western blots demonstrating the expression levels of IL-6, IL-1β, and TNF-α. The quantitative results of IL-6 **(E)**, IL-1β **(F)**, and TNF-α **(G)** are shown in the histogram. NS indicates no significance, n = 6, Multiple group comparisons were made using one-way ANOVA, followed by Bonferroni *post hoc* test, *p < 0.05, **p < 0.01.

Given the key role of the NF-κB signaling pathway in macrophage polarization (Takeda et al., 2011), Western blot analysis was performed to detect the expression and phosphorylation states of NF-κB and IκB-α (Figure 4A). Quantification of the Western blot results indicated that the protein expression of total NF-κB and total IκB-α did not differ between groups, whereas ischemic injury significantly enhanced the phosphorylation of both molecules, which were attenuated by dapagliflozin administration (p-NF-κB/t-NF-κB: HLI vs. HLI + DAPA = 1.45 vs. 1.08; p-IκB-α/t-IκB-α: HLI vs. HLI + DAPA = 1.24 vs. 0.98, one-way ANOVA, *p* < 0.05) (Figure 4B; C). Additionally, inflammatory factors such as IL-1β, IL-6, and TNF-α were measured by Western blot (Figure 4D), and their expression levels were significantly lower in the HLI + DAPA group than in the HLI group (Figure 4E; F, G, one-way ANOVA, *p* < 0.05).

### 3.5 Dapagliflozin treated macrophage conditioned medium promotes proliferation, migration, and tube formation of HUVECs

To further investigate the mechanism by which dapagliflozin promotes angiogenesis, we treated HUVECs under hypoxic conditions with dapagliflozin and observed changes in vascular endothelial cell function *in vitro*. However, we found no significant differences in the migration (Supplementary Figures S3A, B), proliferation (Supplementary Figures S3C, D), and tube formation ability (Supplementary Figures S3E, F, D) of HUVECs between the dapagliflozin-treated and PBS groups.

**FIGURE 5:**
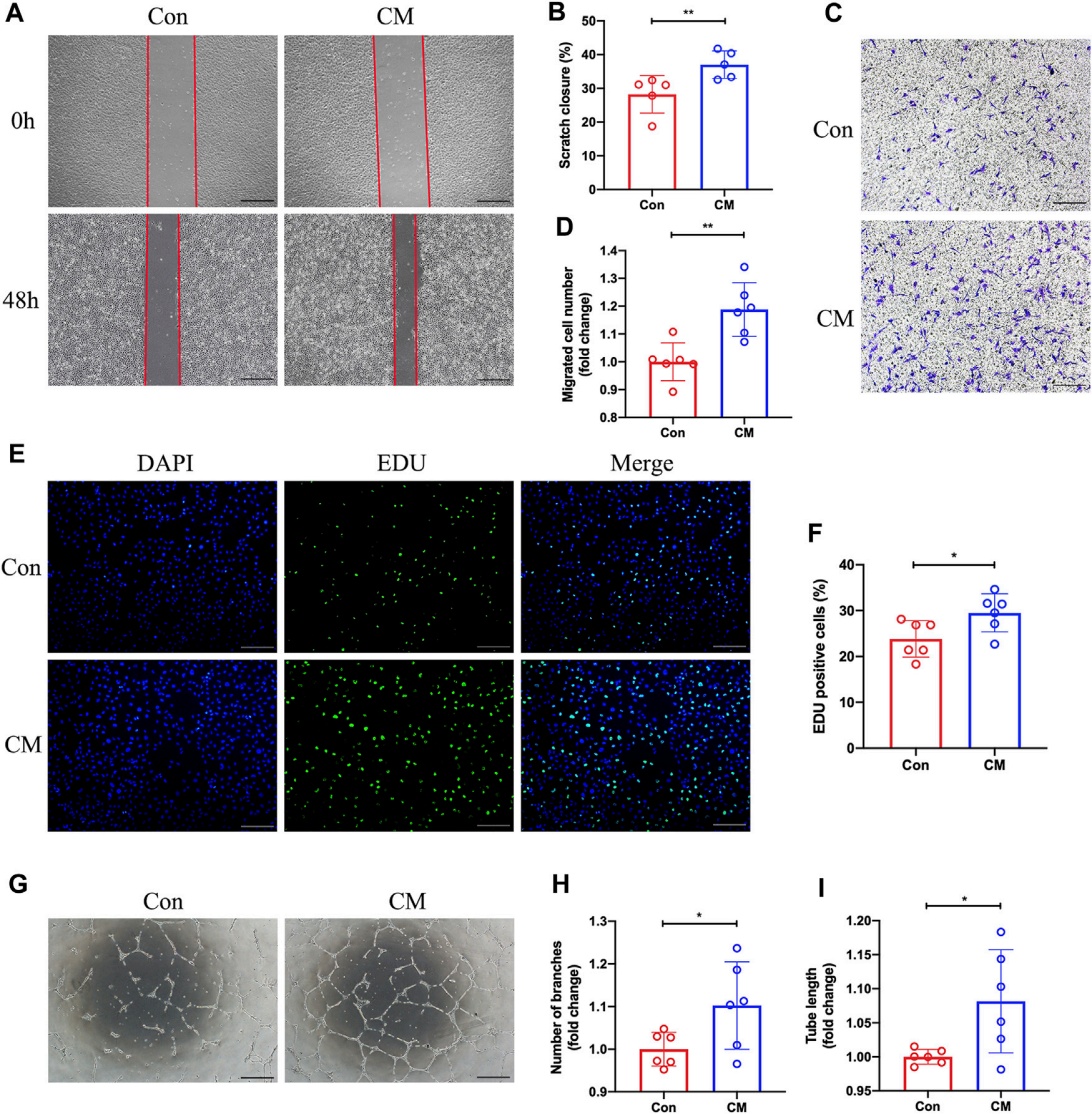
Promotion of HUVECs migration, proliferation, and tube formation by dapagliflozin-treated macrophage CM. Representative images **(A)** of the scratch assay *in vitro* and quantitative analysis **(B)** of the migration area were presented. Scale bar = 500 µm. Representative images **(C)** of the Transwell assay and quantitative analysis **(D)** of the migrated cells were presented to show the HUVECs’ migration ability. Scale bar = 500 µm. **(E)** Representative images of EdU staining showing the effects of the macrophage-CM on the proliferation of HUVECs. Quantification results **(F)** of EdU staining were calculated as the ratio of EdU + cells and DAPI + cells. Scale bar = 200 µm. Representative images **(G)** of the HUVECs tube formation test. The number of branches **(H)** and total length **(I)** of the tubule structure were quantified. Scale bar = 500 µm n = 5 **(A–B)**; n = 6 **(C–I)**, Statistical comparisons were made using unpaired *t*-test, *p < 0.05, **p < 0.01.

We hypothesize that dapagliflozin could indirectly regulate the function of endothelial cells by promoting M2 macrophage polarization. BMDMs were then exposed to DAPA *in vitro* and its conditioned medium was collected, which was subsequently added to HUVECs under hypoxic conditions. Subsequently, scratch assay, transwell, 5-Ethynyl-2′-deoxyuridine (EdU), and tube formation assays were performed. Our scratch test revealed that the CM from DAPA-treated macrophages enhanced the migration ability of HUVECs compared with that from Con group macrophages (Con vs. CM = 28.21% vs. 37.02%, unpaired *t*-test, *p* < 0.01) (Figure 5A; B). Similarly, the CM from DAPA-treated macrophages increased the number of migrated HUVECs in the lower compartment of the transwell chamber (Con vs. CM = 1.00 vs. 1.19, unpaired *t*-test, *p* < 0.01) (Figure 5C; D). Furthermore, DAPA-treated macrophage CM increased the number of EdU-positive cells (Con vs. CM = 23.83% vs. 29.49%, unpaired *t*-test, *p* < 0.05) (Figure 5E; F), indicating its potential to promote HUVEC proliferation. Consistent with these findings, our tube formation assays (Figure 5G) demonstrated that the CM from macrophages treated with DAPA increased the total tube length (Con vs. CM = 1.00 vs. 1.10, unpaired *t*-test, *p* < 0.05) (Figure 5H) and the number of branches (Con vs. CM = 1.00 vs. 1.08, unpaired *t*-test, *p* < 0.05) (Figure 5I).

## 4 Discussion

CLI is a fierce ischemic disease that imposes a significant burden on patients and society due to its association with high amputation rates and mortality rates (Duff et al., 2019). Although various therapeutic modalities are currently employed in clinical practice to enhance blood flow to the ischemic limb, their clinical efficacy remains suboptimal (Teraa et al., 2016). Therapeutic angiogenesis has arisen as a promising therapeutic approach for CLI patients, encompassing gene, protein, drug, and stem cell-based therapies (Veith et al., 2019). Despite their potential, clinical trial results have demonstrated limited efficacy, highlighting the urgent need for agents to induce effective therapeutic angiogenesis.

Dapagliflozin, a selective inhibitor of the sodium-glucose co-transporter protein 2 (SGLT2i), is an effective therapeutic agent for lowering blood glucose levels. Studies have reported that dapagliflozin can alleviate arterial stiffness and endothelial dysfunction in diabetic patients, as well as alleviate dysfunction in non-diabetic endothelial cells (Lee et al., 2018; Cappetta et al., 2020). However, the impact and potential mechanism of dapagliflozin on CLI remain unclear and require further elucidation.

In our research, non-diabetic mice were pretreated with dapagliflozin and induced hindlimb ischemia by ligating the unilateral femoral artery. Laser Doppler imaging assessed perfusion recovery after the operation and found that mice treated with dapagliflozin exhibited faster blood flow recovery compared to the control group, suggesting the potential of dapagliflozin to facilitate blood flow recovery following limb ischemia (Figure 1A-C). In addition, lower necrosis scores and less muscle fibrosis indicate that dapagliflozin can alleviate tissue damage (Figure 1D-F). These results suggest the potential utility of dapagliflozin as a therapeutic agent for CLI.

Subsequently, we applied immunohistochemical assays to assess markers of angiogenesis to determine whether dapagliflozin could reduce limb ischemia injury by promoting angiogenesis. Histological analysis showed that dapagliflozin increased the expression of CD31 and α-SMA in muscle tissue, which are characteristic markers of capillaries and arterioles (Figure 2A-D), suggesting that dapagliflozin may have a role in promoting angiogenesis.

Many studies have shown that macrophages and their polarization status are essential in angiogenesis. In this study, we used M1 and M2 macrophage-specific markers, including iNOS and Arg-1, to identify the effect of dapagliflozin on macrophage polarization through immunofluorescence and WB experiments. *In vivo* and *in vitro*, dapagliflozin could reduce the expression of iNOS while simultaneously increasing the expression of Arg-1, promoting the polarization of macrophages to M2 (Figure 3). The polarization state of macrophages, which can be either proinflammatory (M1) or anti-inflammatory (M2), has significant consequences for tissue damage and repair after ischemia (Locati et al., 2020). M1 macrophages, characterized by the secretion of pro-inflammatory factors and high expression of iNOS, can impair angiogenesis in ischemic tissue, while M2 macrophages, characterized by increased expression of CD206 and Arg-1, can promote tissue repair and angiogenesis (Ganta et al., 2017; Ganta et al., 2019). Previous studies have suggested that dapagliflozin has anti-inflammatory effects, which have also been demonstrated in clinical trials (Chen et al., 2020; Abdollahi et al., 2022). These results provide further evidence of the anti-inflammatory effects of dapagliflozin and its prospect as a therapeutic agent for ischemic conditions.

Further investigation was conducted to clarify the potential mechanism underlying the macrophage polarization induced by dapagliflozin. Previous investigation has proposed that the NF-κB signal pathway plays a pivotal role in macrophage polarization, and its activation promotes the polarization of macrophages to M1 (Li et al., 2020). In this study, we employed WB to measure the total protein levels and phosphorylation states of NF-κB and IκB, and our findings demonstrated that dapagliflozin treatment reduced the activation of the NF-κB signaling pathway (Figure 4). Under resting conditions, NF-κB exists in the cytoplasm as a dimer, whereas phosphorylation of IκB leads to the separation of the NF-κB subunit from the dimer, resulting in its further stimulation through phosphorylation. Upon activation, the NF-κB subunit translocates to the nucleus, where it functions as a transcription factor (Hayden and Ghosh, 2008; Oh et al., 2019). In summary, these findings suggest that dapagliflozin can modulate the polarization state of macrophages, at least in part, through the inhibition of the NF-κB signaling pathway. The study by Tsung-Ming Lee et al. also found that dapagliflozin could regulate macrophage polarization status by affecting STAT3 (Lee et al., 2017).

Angiogenesis is a complex process that relies on the multiple functions of endothelial cells, including their ability to proliferate, migrate, and form tubes (van der Kwast et al., 2019; Pérez-Cremades et al., 2020). To this end, we investigated the impacts of dapagliflozin on endothelial cells *in vitro*. Our findings are consistent with prior studies, demonstrating that dapagliflozin does not directly affect the proliferation, migration, or tube formation in HUVECs (Behnammanesh et al., 2019). However, the functions mentioned above were enhanced when HUVECs were exposed to a macrophage-conditioned medium treated with dapagliflozin. These observations suggest that dapagliflozin indirectly enhances the proliferation, migration, and tubular formation potential of HUVECs by promoting the polarization of macrophages to M2 (Figure 5). Nugrahaningrum et al. found that intramuscular injection of dapagliflozin promoted paracrine function in skeletal muscle cells after lower limb ischemia in diabetic mice, which in turn enhanced the proliferative and migratory potential of vascular endothelial and smooth muscle cells and induced angiogenesis (Nugrahaningrum et al., 2020). Nonetheless, the underlying mechanism requires further investigation.

There are some shortcomings in this study. Firstly, angiogenesis involves a complex series of processes culminating in the formation of a mature vascular system (Potente et al., 2011). Although this study demonstrated that dapagliflozin accelerated the restoration of blood perfusion and increased the density of capillaries and arteriole in HLI mice, vascular permeability was not further examined. Secondly, our study centered on the classical NF-κB signaling pathway due to its integral role in macrophage polarization and angiogenesis. However, we acknowledge the presence of other signaling pathways like STAT3, ERK, and others that warrant investigation in future studies. Finally, angiogenesis is a process that involves different cell types. For example, pericytes promote vascular maturation and stabilization and communicate directly with endothelial cells. Furthermore, the absence of pericytes has been associated with increased vascular permeability (Caporali et al., 2015). Regrettably, the present study focused primarily on the interactions between macrophages and endothelial cells, potentially missing out on other crucial cell types that may play significant regulatory roles in the process of angiogenesis, such as skeletal muscle cells or pericytes.

## Conclusion

**FIGURE 6:**
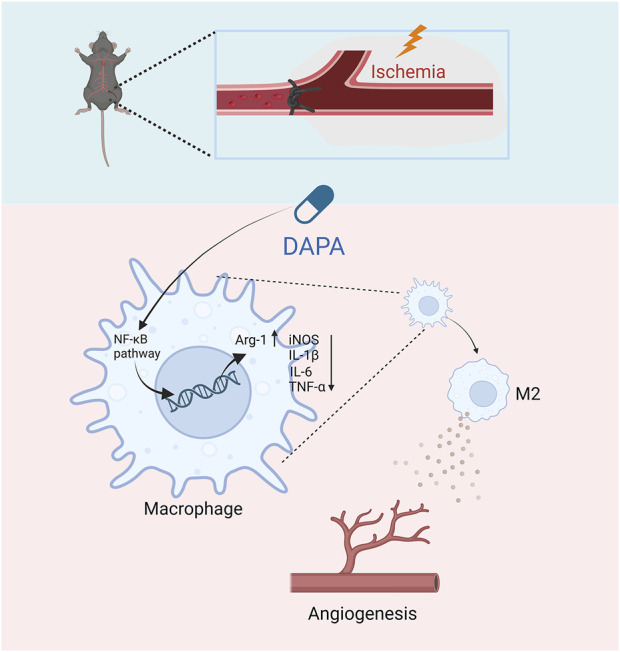
The schematic diagram of the major study findings shows how dapagliflozin promoted angiogenesis in mice with hindlimb ischemia. Dapagliflozin may inhibit the NF-κB signaling pathway of macrophages in ischemic muscle, thereby inhibiting the excessive secretion of inflammatory factors by macrophages and promoting the transformation of M2-type macrophages. Afterward, M2 macrophages promote ischemic tissue repair and promote angiogenesis.

In summary, this study evaluated the effects of dapagliflozin on the post-ischaemic limb by establishing an HLI mouse model, and the results showed that dapagliflozin could enhance angiogenesis in the ischemic muscle tissues of non-diabetic mice via macrophage targeting. Specifically, dapagliflozin may stimulate macrophage polarization from the M1 to M2 phenotype by inhibiting the NF-κB pathway, thereby indirectly improving endothelial cells’ proliferation, migration, and tube formation ability (Figure 6). Our research suggests that dapagliflozin may be a potential medicinal agent for ischemic disorders while providing a novel therapeutic option for treating CLI.

## Data Availability

The raw data supporting the conclusions of this article will be made available by the authors, without undue reservation.
