# Supplementary material for: Dapagliflozin promotes angiogenesis in hindlimb ischemia mice by inducing M2 macrophage polarization
Source: Front Pharmacol. 2023 Sep 21;14:1255904. doi: 10.3389/fphar.2023.1255904 (PMC10558177; doi:10.3389/fphar.2023.1255904)
Supplement: Supplementary file 1 [file DataSheet1.docx]

Supplementary Material

# Supplementary Figures

# **
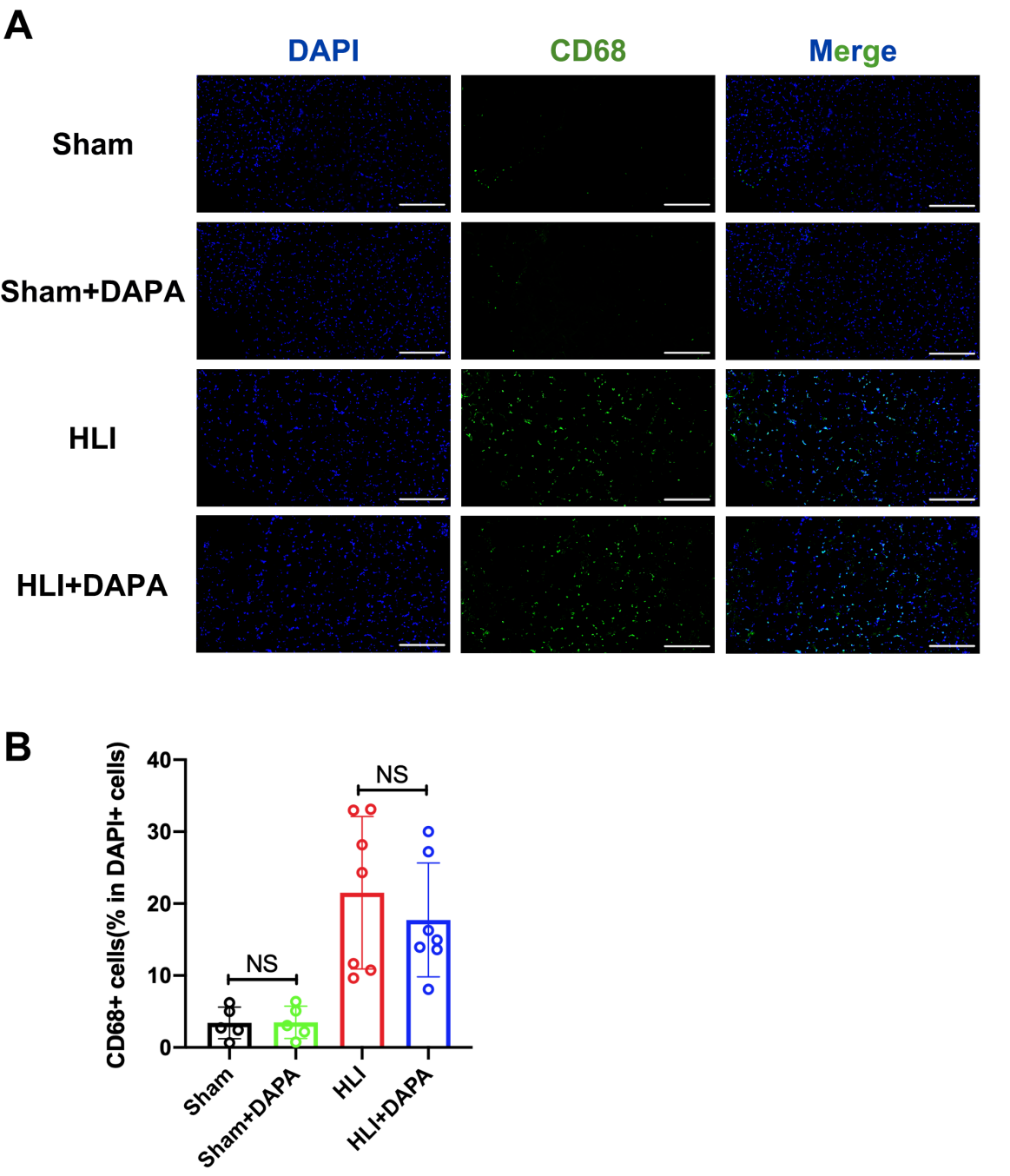
**

**Supplementary Figure 1.** The number of macrophages in the ischemic muscle tissue of HLI mice was not altered by DAPA treatment. (A) Representative images of CD68 and DAPI staining in the ischemic gastrocnemius muscle. (B) Quantitative analysis of the total macrophages was calculated as the ratio of CD68+ cells and DAPI+ cells. Scale bar =100μm. NS indicates no significance. n=5-7, Multiple group comparisons were made using one-way ANOVA, followed by Bonferroni post hoc test.

**
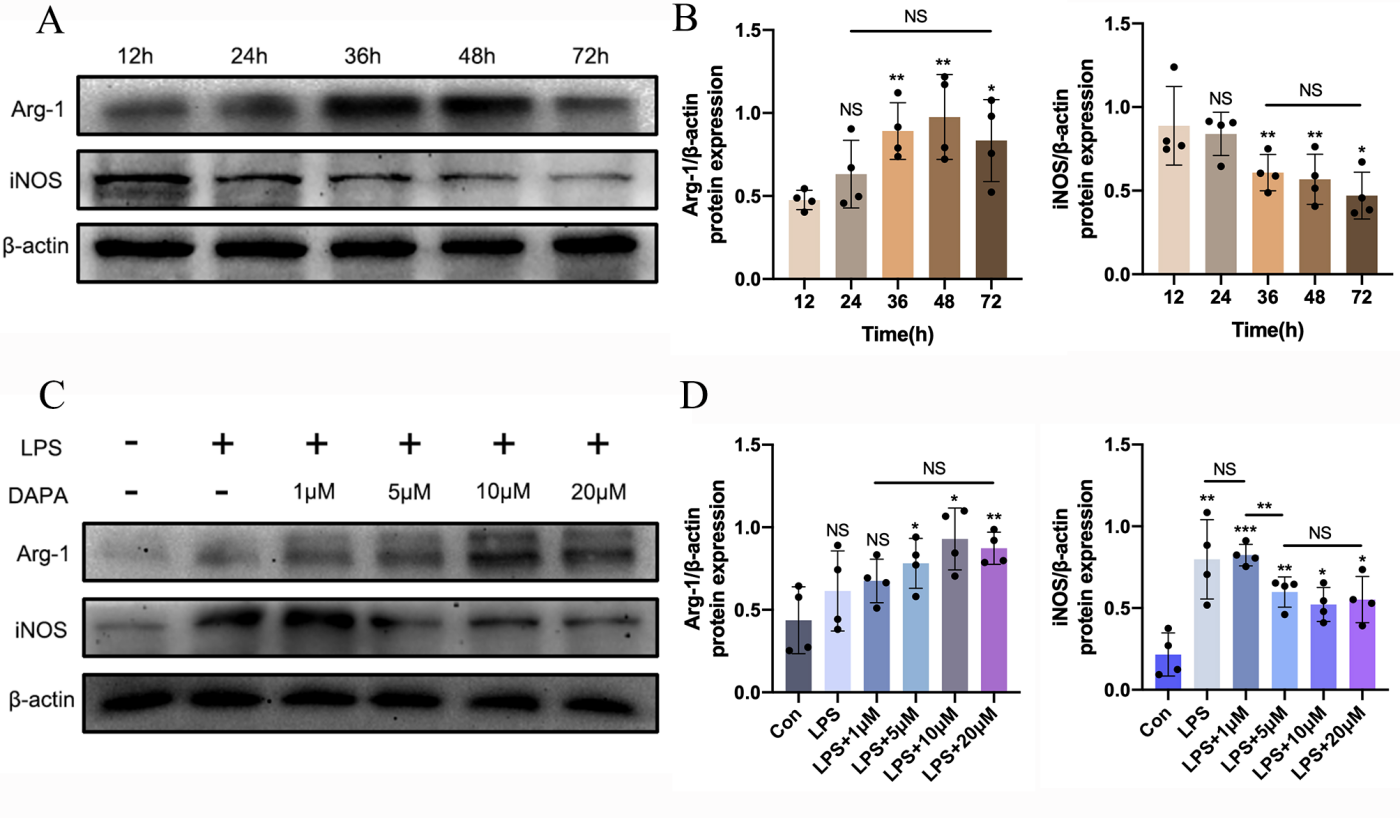
**

**Supplementary Figure 2.** The effect of dapagliflozin on the polarization of BMDMs under different treatment times and concentrations *in vitro*. (A and B) Representative western blotting images (A) and summary densitometry graph (B) illustrating expression of Arg-1 and iNOS in LPS-stimulated mouse BMDMs treated with dapagliflozin at different times *in vitro*. (C and D) Representative western blotting images (C) and summary densitometry graph (D) illustrating expression of Arg-1 and iNOS in mouse BMDMs treated with different concentrations of dapagliflozin. NS indicates no significance, n=4, Multiple group comparisons were made using one-way ANOVA, followed by Bonferroni post hoc test, *P<0.05, **P<0.01.

**
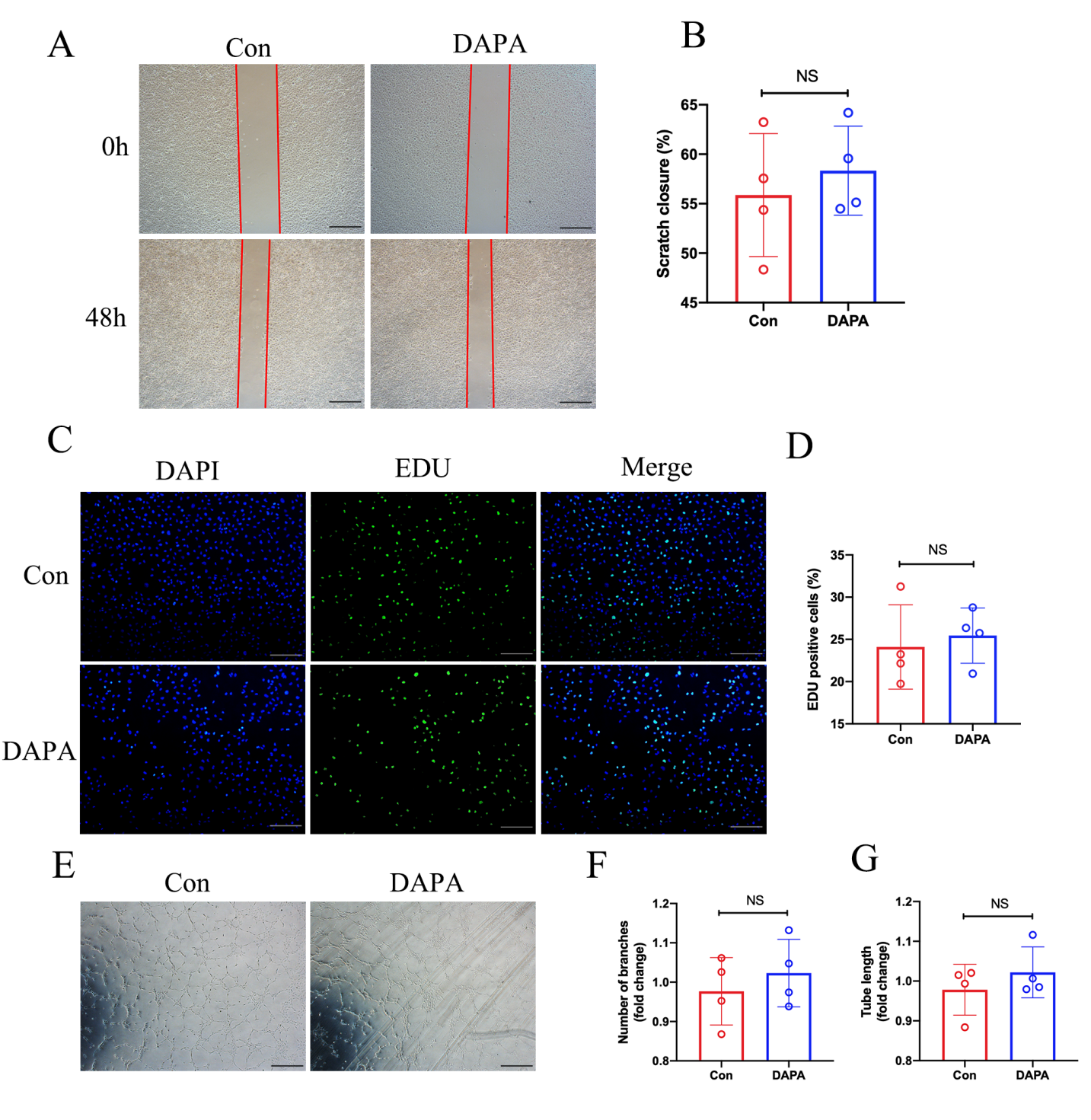
**

**Supplementary Figure 3.** Direct treatment of dapagliflozin does not change the ability of migration, proliferation, and tube formation of HUVEC. Representative images (A) of the scratch assay *in vitro* and quantitative analysis (B) of the migration area were presented. Scale bar =500µm. (C) Representative images of EdU staining showing the effects of the macrophage-conditioned medium on the proliferation of HUVECs. Quantification results (D) of EdU staining were calculated as the ratio of EdU+ cells and DAPI+ cells. Scale bar =200µm. Representative images (E) of the HUVECs tube formation test. The number of branches (F) and total length (G) of the tubule structure were quantified. Scale bar =500µm. NS indicates no significance. n=4, Statistical comparisons were made using Mann–Whitney test.
